# A Decade of Th9 Cells: Role of Th9 Cells in Inflammatory Bowel Disease

**DOI:** 10.3389/fimmu.2018.01139

**Published:** 2018-05-24

**Authors:** Shachi Pranjal Vyas, Ritobrata Goswami

**Affiliations:** School of Bioscience, IIT Kharagpur, Kharagpur, India

**Keywords:** T helper cells, cytokines, Th9 cells, IL-9, inflammatory bowel disease, ulcerative colitis, claudin, occludin

## Abstract

T helper cell subsets play a critical role in providing protection against offending pathogens by secreting specific cytokines. However, unrestrained T helper cell responses can promote chronic inflammation-mediated inflammatory diseases. Dysregulated T helper cell responses have been suggested to be involved in the pathogenesis of multiple inflammatory diseases, including allergic airway inflammation, rheumatoid arthritis, and inflammatory bowel disease (IBD) among others. Aberrant pro-inflammatory responses induced by Th1, Th2, and Th17 subsets are known to trigger IBD. IBD is a chronic inflammatory disease characterized by weight loss, diarrhea, pain, fever, and rectal bleeding. It poses a major health burden worldwide owing to the increased risk of colorectal cancer development. Despite numerous therapeutic advancements, IBD still remains a major health burden due to the inefficiency of the conventional therapies. Recently, IL-9-secreting Th9 cells are known to be involved in the pathogenesis of IBD. However, the role of Th9 cells and their secretory cytokine IL-9 in IBD is unclear. The functional relevance of Th9 cells is also relatively understudied in IBD. Thus, investigating the actual role of various T helper cell subsets including Th9 cells in IBD is essential to develop novel therapies to treat IBD. Here, we highlight the role of Th9 cells in promoting IBD. We discuss the mechanisms that might be employed by Th9 cells and IL-9 in promoting IBD and thereby propose potential targets for the treatment of Th9 cell-mediated IBD.

## Introduction

T helper cells (CD4^+^ T cells) constitute one of the key components of adaptive immune system. They play a crucial role in imparting protective immunity against a wide range of pathogens by secreting specific cytokines and chemokines (1). CD4^+^ T cells attain the potential to secrete specific cytokines in response to environmental signals including strength of TCR-peptide engagement, co-stimulatory signaling, cytokine signals and expression of transcription factors in a process known as T helper cell differentiation (2, 3). Differentiated T helper cells play a myriad of functions in obliterating the infecting pathogens. However, when these cell-mediated responses are not properly regulated, unrestrained CD4^+^ T cell responses lead to chronic inflammation and tissue damage. Exactly one decade back, another subset of CD4^+^ T cells, which predominantly secrete the pro-inflammatory cytokine IL-9 was identified and christened as Th9 cells (4). Th9 cells have been known to provide immunity against helminth parasites (4–6). Moreover, they also play a vital role *in vivo* by providing antitumor immunity by secreting cytokines such as IL-9, IL-3, and IL-21 (7). Interestingly, Th9 cells are also known to induce inflammation and thereby exacerbate inflammatory diseases, including allergic asthma, multiple sclerosis, rheumatoid arthritis, among others (8, 9). Recent studies suggest that Th9 cells and their secretory cytokine IL-9 can also promote inflammatory bowel disease (IBD), which poses a significant risk factor for colon cancer development (10). However, the functional relevance of Th9 cells in IBD remains underappreciated and needs to be further investigated. Here, we briefly discuss how Th9 cells may be critical for the development of IBD.

## Th9 Cells in Inflammation: Role in IBD

Th9 cells are known to induce pathogenic responses in numerous inflammatory diseases including IBD, rheumatoid arthritis, allergic asthma among others. In this section we discuss the potential role of Th9 cells in IBD. IBD is a gastrointestinal tract disorder arising due to unrestrained gut inflammation and is divided into Crohn’s disease (CD) and ulcerative colitis (UC) (11). Despite numerous therapeutic advancements, many patients continue to suffer from the complications of IBD due to lack of proper understanding of key immune players responsible for the pathogenesis of IBD.

Hallmark of IBD is chronic inflammation of the gut (9). Several T helper cell subsets are known to promote inflammatory responses in the gut. Previous studies have indicated that Th1 and Th2 cells are responsible for the pathogenesis of CD and UC, respectively (12). Moreover, Th17 cells were also demonstrated to induce chronic intestinal inflammation and promote IBD by secreting IL-17A (13). Additionally, it also known that in CD, Th17 cells secrete key signature cytokines of other T helper cell subsets such as IFN-γ (Th1) and IL-4 (Th2) resulting in the development of pathogenic Th17/Th1 or Th17/Th2 phenotype. Th17/Th1 cells are known to be more pathogenic than Th17 cells and are a promising target for the treatment of inflammatory conditions including CD (14, 15). Recently, it was observed that the transfer of Th9 cells resulted in the aggravation of UC in the gut mucosa of RAG-deficient mice indicating a crucial role of Th9 cells in IBD progression (16). Moreover, a correlation between UC progression and IL-9 secreted by Th9 cells in UC patients has also been demonstrated recently (16, 17).

Several murine models of chronic inflammation have been generated to understand the role of various immune players in IBD pathogenesis (18, 19). In TNBS-induced colitis model, there was an increase in the number of PU.1-expressing T cells and IL-9 secretion in the intestinal epithelial cells; underlying the importance of the transcription factor PU.1 in Th9 cell development (2, 20). IL-9 induced inflammation in mucosal epithelial cells and promoted colitis upon treatment with TNBS (20). Moreover, IL-9 deficiency resulted in reduced number of PU.1^+^ T cells and protected mice from colitis in TNBS-colitis model indicating a role of Th9 cells and their secretory cytokine in the regulation of mucosal inflammation-mediated colitis (20). In oxazolone-mediated colitis model also there was an increase in the expression of IL-9 and IL-9R by intestinal epithelial cells (16). Moreover, deficiency of PU.1^+^ IL-9^+^ T cells resulted in suppression of experimental colitis upon oxazolone treatment indicating a critical role of PU.1^+^ T cells in promoting UC in mice. The role of IL-9 in promoting UC was also corroborated by investigating the intestinal mucosa of UC patients and healthy volunteers. There was enhanced number of mucosal T cells that express PU.1. Moreover, abundant IL-9R expressing intestinal epithelial cells were observed in the gut mucosa of UC patients indicating the role of IL-9 and Th9 cells in promoting colitis (16). These indicate that IL-9^+^, PU.1^+^ Th9 cells play a vital role in the progression of UC and their differentiation needs to be tightly regulated to prevent the disease progression. Interestingly, a contradictory observation was demonstrated in DSS-induced model of UC, wherein IL-9 secretion by invariant natural killer T (iNKT) cells resulted in resolution of intestinal inflammation instead of promoting inflammation (21). Overall these studies suggest that the role of IL-9 in disease outcome depends on both the cytokine and the source of its secretion.

The potential mechanism for Th9-mediated colitis has been unraveled by investigating the function of Th9 cells and IL-9 in regulating the expression of tight junction proteins essential for maintaining intestinal barrier integrity (20). Tight junction proteins including claudins and occludin are essential for maintaining intestinal barrier functions and alteration in their expression is known to be responsible for numerous inflammatory disorders (22). In IL-9 knockout mice treated with TNBS, claudin 1 expression was downregulated; however, the expression of claudin 2 remained unchanged. Interestingly, in oxazolone colitis model, there was a downregulation in the expression of claudin 2 in IL-9-deficient mice than wild-type mice. This indicates that IL-9 might disrupt intestinal permeability by enhancing the expression of claudin 2 in oxazolone-mediated colitis (20). Thus, it could be suggested that IL-9 promotes colitis by regulating the expression of different tight junction molecules in different inflammatory conditions (20). Occludin, another tight junction molecule is also known to be upregulated in UC patients, but the role of IL-9 in regulating the expression of occludin in UC patients is not known so far (23). Disruption of intestinal barrier by IL-9 resulted in enhanced bacterial translocation in the mucosa of wild-type mice than IL-9-deficient mice in oxazolone-colitis model. This suggests that the potential mechanism for IL-9-mediated colitis involves disruption of the intestinal barrier culminating in increased bacterial entry into the mucosa and associated pro-inflammatory responses (16). The role of Th9 cells in regulating colitis in various mice models have been mentioned in Table 1.

**Table 1:** Mechanisms employed by Th9 cells in regulating ulcerative colitis in various mice models.

Colitis model	Mechanism	Reference
Oxazolone-induced colitis	Upregulation of claudin 2 expression	(16)
TNBS-induced colitis	Upregulation of clauidn 1 expression	(16)
DSS-induced colitis	Resolution of inflammation	(21)

IL-9 secreted by Th9 cells could be directly or indirectly involved in the progression of IBD. IL-9 is a pleiotropic cytokine involved in the activation and migration of various immune cells to the site of inflammation by inducing the production of chemokines. IL-9 has been reported to augment the expression of the chemokine eotaxin in smooth muscle cells (24). IBD has been associated with the expression of multiple chemokines including RANTES, CCL19, and CCL21 (25). Thus, IL-9 produced by Th9 cells might also possibly induce the expression of such chemokines in intestine during IBD to promote inflammatory responses. It is also possible that the *in vivo* effects of IL-9 in IBD could depend on other immune cells and their secretory cytokines. IL-9 secreted by Th9 cells is known to promote Th2-skewed immune responses, thereby inducing UC in the process (16). IL-9 is also known to recruit and activate mast cells which secrete histamine, pro-inflammatory cytokines, and mast cell proteases to the intestine resulting in increased intestinal permeability and intestinal anaphylaxis (26). Thus, it is possible that IL-9 might recruit mast cells to intestinal epithelial cells, which in turn alter the intestinal barrier culminating in inflammation in the gut and UC.

Moreover, there is lack of evidence regarding the plasticity of Th9 cells in IBD. Th9 cells could possibly switch to an intermediate Th17 or Th1 cell type that would lead to the inflammation. Thus, blocking either Th17 or Th1-specific cytokine in such conditions could be of therapeutic interest. Therefore, it is critical to determine the actual role of Th9 cells and their secretory cytokine IL-9 in IBD.

## What Lies Ahead in the Future: Therapies Targeting Cytokines Signaling in Intestinal Mucosa for Improved Treatment Outcomes?

**Figure 1:**
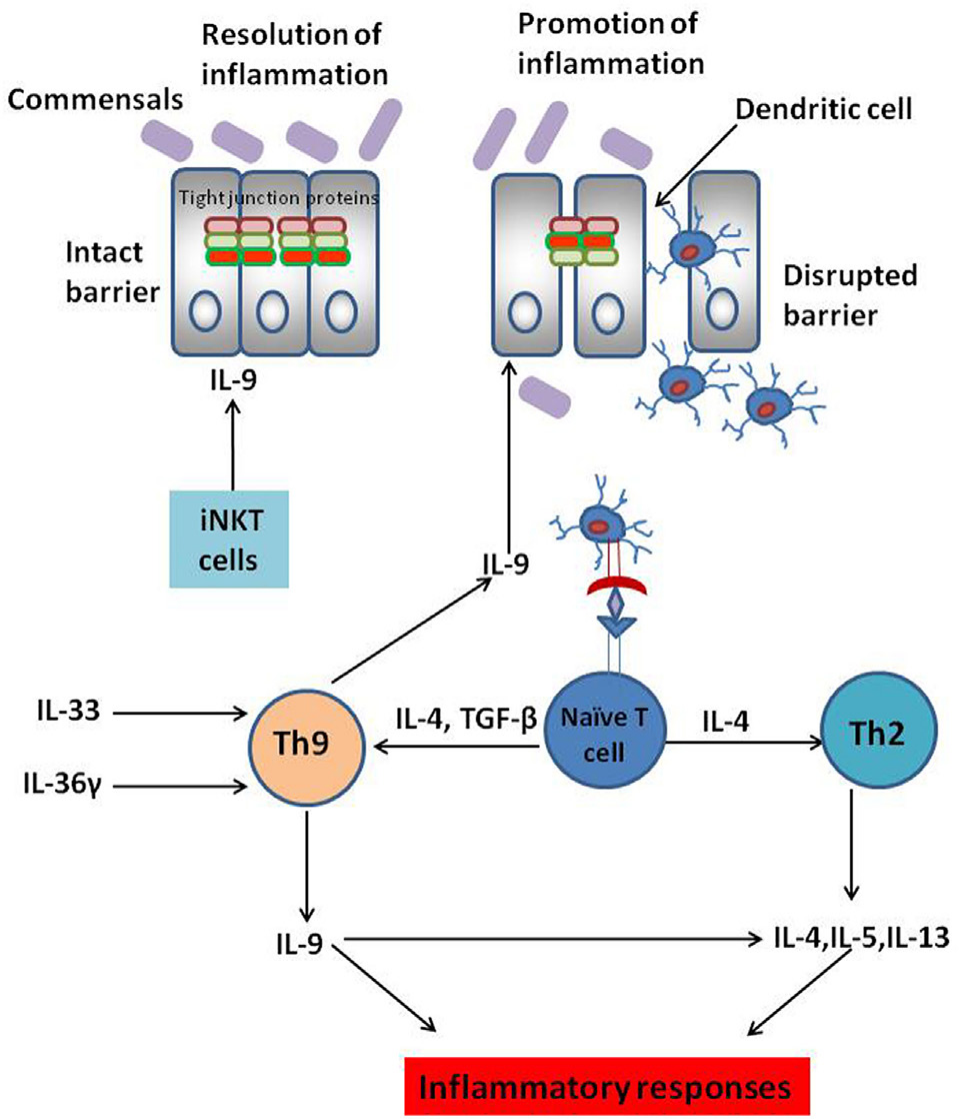
Complex role of IL-9-secreting Th9 cells in inflammatory bowel disease (IBD). Interleukin-9, a pleiotropic cytokine, is known to play dichotomous role in IBD. IL-9 secretion by Th9 cells disrupts intestinal barrier permeability resulting in the entry of innocuous antigens into the gut mucosa. This leads to antigen presentation by the dendritic cells to naïve CD4^+^ T cells culminating in T helper differentiation. Several cytokines are known to promote Th9 cell differentiation. IL-33 and IL-36 play a key role in Th9 cell proliferation in the gut mucosa culminating in aggravated colitis. IL-9 secreted by Th9 cells is also known to promote Th2-like responses culminating in the progression of ulcerative colitis (UC). However, IL-9 secreted by cells other than Th9 cells such as invariant natural killer T (iNKT) cells are known to dampen inflammatory responses rather than promoting them. Thus, anti-IL-9 antibody might not be the “magic bullet” to treat IBD. Instead, targeting the cytokines involved in Th9 cell expansion in the gut mucosa could be a possible strategy to maintain optimal Th9 cell development in the gut and thereby restrain Th9 cell-mediated UC.

The actual role of IL-9 secreting Th9 cells in various inflammatory conditions is not clear. The role of IL-9 in various inflammatory conditions also depends on the *in vivo* mice models. In OVA-induced model of allergic inflammation, Th9 cells were the main source of IL-9; while, in papain model of allergic inflammation ILC2s were the key source of IL-9 (2). Since observations from T cell transfer, TNBS and oxazolone-induced UC indicate a pathogenic role of IL-9 secreted by Th9 cells in the progression of UC; it would be benefical to determine whether UC progression can be curbed by preventing the expansion of Th9 cells in gut. Several cytokines and signaling molecules are known to promote Th9 cell development and proliferation. A recent study has demonstrated that Th9 cell differentiation can be promoted by IL-36γ (IL-1 family cytokine) signaling (27). In oxazolone-induced colitis, IL-36γ signaling was observed to promote naïve CD4^+^ T cell differentiation to Th9 cells by inducing phosphorylation of STAT5 and STAT6. Moreover, IL-36γ secretion also impaired Treg cell differentiation (27). IL-36γ signaling, therefore, abrogates Treg cell development and alters the balance between Th9 and Treg cells resulting in aggravated intestinal inflammation and progression of IBD (27). Thus, targeting IL-36 binding with its receptor in intestinal mucosal cells might be a promising strategy to control Th9 cell-mediated colitis (27). It has also been demonstrated that there is an increase in the production of IL-33 in UC patients. IL-33 is involved in promoting Th9 cell development independent of IL-4 signaling and blocking IL-33 could abrogate Th9 cell development (28, 29). Thus, these signaling molecules and cytokines could serve as potential targets for regulating Th9 cell differentiation and associated diseases. The potential mechanisms for regulating Th9 cell differentiation and IL-9 secretion have been depicted in Figure 1.

## Future Directions

The role of IL-9 in propagating UC is complex. IL-9 is known to prevent the complications of UC disease by suppressing the secretion of IFN-γ and IL-17A (21). Moreover, IL-9 secreted by iNKT cells in the DSS-mediated colitis model also dampened the immune response by inducing the release of IL-10 and TGF-β (anti-inflammatory cytokines) (21). Therefore, further studies involving determination of the source of IL-9 secretion in UC could potentially improve the therapeutic outcome among UC-infected patients. It has been observed that neutralization of IL-9 inhibits intestinal inflammation in oxazolone-induced colitis model (16). However, it is not known whether blockade of IL-9 in other mice models of colitis would also give similar results or not. Moreover, the effect of IL-9 neutralization in humans is also unknown. Thus, deficiency of IL-9 might aid in controlling inflammation in UC, but it might also impair the protective immunity imparted by IL-9 secretion. Therefore, due to contradictory observations using various mice models of IBD, there is an unmet need to determine the actual role of Th9 cells and IL-9 in humans suffering from IBD. Additionally, it is essential to unravel the mechanisms employed by IL-9 secreting Th9 cells in the progression of IBD to develop novel therapeutic strategies and thereby curb intestinal inflammation and IBD.

## Author Contributions

SV wrote the manuscript. RG conceptualized the idea and co-wrote the manuscript.

## Conflict of Interest Statement

The authors declare that the research was conducted in the absence of any commercial or financial relationships that could be construed as a potential conflict of interest.
